# Deep learning based rapid X-ray fluorescence signal extraction and image reconstruction for preclinical benchtop X-ray fluorescence computed tomography applications

**DOI:** 10.1038/s41598-025-03900-0

**Published:** 2025-06-04

**Authors:** Amrit Kaphle, Sandun Jayarathna, Sang Hyun Cho

**Affiliations:** 1 Department of Radiation Physics, The University of Texas MD Anderson Cancer Center, Houston, TX 77030 USA; 2 Department of Radiation Physics, Department of Imaging Physics, The University of Texas MD Anderson Cancer Center, Houston, TX 77030 USA

**Keywords:** Biomedical engineering, Imaging

## Abstract

Recent research advances have resulted in an experimental benchtop X-ray fluorescence computed tomography (XFCT) system that likely meets the imaging dose/scan time constraints for benchtop XFCT imaging of live mice injected with gold nanoparticles (GNPs). For routine in vivo benchtop XFCT imaging, however, additional challenges, most notably the need for rapid/near-real-time handling of X-ray fluorescence (XRF) signal extraction and XFCT image reconstruction, must be successfully addressed. Here we propose a novel end-to-end deep learning (DL) framework that integrates a one-dimensional convolutional neural network (1D CNN) for rapid XRF signal extraction with a U-Net model for XFCT image reconstruction. We trained the models using a comprehensive dataset including experimentally-acquired and augmented XRF/scatter photon spectra from various GNP concentrations and imaging scenarios, including phantom and synthetic mouse models. The DL framework demonstrated exceptional performance in both tasks. The 1D CNN achieved a high coefficient-of-determination (R² > 0.9885) and a low mean-absolute-error (MAE < 0.6248) in XRF signal extraction. The U-Net model achieved an average structural-similarity-index-measure (SSIM) of 0.9791 and a peak signal-to-noise ratio (PSNR) of 39.11 in XFCT image reconstruction, closely matching ground truth images. Notably, the DL approach (vs. the conventional approach) reduced the total post-processing time per slice from approximately 6 min to just 1.25 s.

## Introduction

X-ray fluorescence (XRF) analysis involves irradiating a sample with X-rays, inducing the emission of characteristic XRF photons, and detecting and analyzing these XRF photons, which altogether enable non-invasive determination of elemental composition within the irradiated sample^1,2^. XRF analysis can be combined with XRF mapping or computed tomography (CT) techniques for quantitative imaging of the elemental distribution within the sample^3,4^. Given this unique capability, both XRF-based imaging approaches, originally developed with monochromatic synchrotron X-ray beams and typically known as direct XRF imaging (or XFI) and XRF CT (or XFCT), respectively, can be applied for imaging of both exogenous and endogenous metallic elements present within biological samples and imaging objects. In principle, these modalities, in conjunction with traditional contrast agents (e.g., iodine) or metal nanoparticles (MNPs) such as gold nanoparticles (GNPs) targeted for specific biological makers, also allow for molecular imaging that offers distinct advantages (e.g., no requirement of radiotracers) compared with conventional molecular imaging modalities such as positron emission tomography (PET) and single photon emission CT (SPECT). Over the years, synchrotron-based XFI has been applied for mapping of endogenous iodine distribution in murine thyroids^5^, exogenous iodine-based agent tracking immune cells in mice, and exogenous GNP distribution in murine spinal cords^6^. Synchrotron-based XFCT has also been applied for quantitative imaging of iodine-based agent uptake in mice (e.g., uptake of an iodine-labeled perfusion agent in mice brain^7^.

While advantageous, especially in terms of XRF production and detection, due to the use of monochromatic synchrotron X-ray beams^8^, synchrotron-based XFCT/XFI has some limitations (e.g., limited accessibility) for widespread biomedical applications. To overcome such limitations, numerous studies^9–12^ have been conducted over the years to show that XFCT/XFI, XFCT in particular, can be implemented on a benchtop setting (“benchtop XFCT”) for the purpose of routine whole-body imaging of small animals injected with GNPs using an ordinary polychromatic X-ray source. These studies have shown that benchtop XFCT has the ability to accurately localize/quantify GNPs present within small animal–sized objects by acquiring gold K-shell XRF photons emitted from the GNPs using commercially available energy-resolving single-crystal cadmium telluride (CdTe) detectors. As reported in our more recent studies^13,14^, our experimental benchtop cone-beam XFCT system adopting a single crystal CdTe detector allowed for the detection of biologically relevant low concentration of GNPs (e.g., 0.02–0.03% by weight (wt%)) present in a small animal-sized phantom using ~ 2 cGy of X-ray dose (or 10 s of irradiation time) per projection. Since such an experimental system typically requires a shifting of the detector to acquire full projection data, the overall X-ray dose and scan time, depending on the number of projections, may exceed the level deemed acceptable (e.g., ≤ 40 cGy and 1 h) for routine in vivo imaging^8^. Despite this type of difficulty with the system adopting a single-crystal detector (& other technical issues with the system adopting array/pixelated detectors to be explained below), there have been attempts to demonstrate benchtop XFCT/XFI-based in vivo/ex vivo quantitative imaging of both passively and actively targeted MNPs such as molybdenum NPs^15^, gadolinium NPs^16^, and GNPs^11,17–19^ accumulated in mice organs and xenograft tumors.

One strategy to address the aforementioned challenge of benchtop XFCT for routine in vivo imaging is to simultaneously collect XRF signals using a custom array of single-crystal detectors or pixelated detectors, which can be implemented through hardware upgrading. As demonstrated in previous experimental studies^17,20–22^, this strategy can result in significantly less scan time and X-ray dose, making the overall process acceptable for routine in vivo imaging. Despite such benefit, it also poses new challenges that need to be successfully dealt with for its application to routine in vivo imaging. Setting aside hardware-specific issues (e.g., decreased energy resolution of a pixelated detector, compared to a single-crystal detector), seemingly the most critical challenge is the current inability for speedy handling (on a real-time basis) of XRF signal extraction from massive amount of spectral data (acquired from 2D pixelated detectors) and XFCT image reconstruction. Traditional methods^10,11,23,24^ for XRF signal extraction and XFCT image reconstruction involve complex and time-consuming spectral data postprocessing procedures. These methods often involve peak fitting, background subtraction, attenuation correction, and spectral deconvolution, which can be computationally expensive, time consuming, and sometimes even noise-sensitive. For XFCT image reconstruction, filtered back-projection (FBP) has been the conventional choice due to its computational efficiency. However, FBP tends to compromise image quality, especially in spatial resolution and low-contrast detectability. While iterative reconstruction methods, such as maximum-likelihood expectation maximization (MLEM)^9,25^, ordered subsets expectation maximization (OSEM)^26^, can potentially produce more accurate images through iterative projection and back-projection, they are computationally demanding and time-consuming. Similar to FBP, these iterative approaches also struggle to preserve low-contrast details^27^. In addition, for routine preclinical applications, it is crucial not only to enhance the XFCT image quality but also to aim for the real-time display of reconstructed XFCT images, a capability that remains virtually unattainable with the current technology.

One potential solution to the problems mentioned above is the integration of advanced deep learning (DL) approaches using convolutional neural network (CNN), which may enable almost real-time XRF signal extraction and XFCT image reconstruction. The CNN is capable of extracting diverse features from low to high levels, which is important for superiority in pattern recognition performance. More recently, the CNN has got state-of-the-art performance on many medical imaging problems^28,29^. A detailed review of literature has shown that end-to-end approaches for XRF signal extraction and XFCT image reconstruction using DL methods are seldom reported. While DL methods^30–32^ have demonstrated remarkable success in various image reconstruction and signal extraction tasks, its application to XFCT has been relatively unexplored. This is primarily due to the complex nature of data used for XFCT, which requires handling of both spectral and tomographic information within a single framework. Additionally, the lack of large well-annotated datasets and standardized validation methods has limited the development of fully integrated DL solutions in XFCT. For signal extraction, one-dimensional (1D) CNNs have demonstrated remarkable efficacy across diverse domains including near-infrared spectroscopic calibration^33^, estimation of soil organic carbon contents through spectroscopic modeling^34^, and prediction of mixture component concentrations from Raman spectra^35^. These applications emphasize the versatility of 1D CNNs in extracting meaningful features from spectral data. Conversely, the majority of DL-based research in imaging has concentrated on conventional modalities like CT/PET/SPECT, using DL architectures such as U-Net^29,36^, GAN^37^, and FBPConvNet^30^. However, the direct transferability of these models to the unique characteristics of XFCT is limited. Preliminary studies by Eom et al.^38^ have utilized 1D CNNs for Compton background reduction in XRF spectra. Also, Jung et al.^39^ and Gao et al.^40^ have employed U-Net for Compton background elimination and self-absorption correction in reconstructed images, respectively. Unlike conventional methods that rely on fixed background subtraction, DL-based approach is trained on spectra with varying signal-to-background ratios (with and without elemental XRF peak presence), enabling it to extract meaningful signals even when statistical significance is marginal. This is particularly advantageous near the detection limit, where traditional techniques often struggle. Nevertheless, a comprehensive end-to-end model incorporating both XRF signal extraction and XFCT image reconstruction remains elusive. Moreover, the XRF signal extraction process in XFCT is often treated as a separate postprocessing step, neglecting the potential benefits of integrating it with the image reconstruction pipeline. XRF spectra for benchtop XFCT are inherently complex, characterized by low signal-to-noise ratio, Compton scatter overlap, and attenuation. Thus, these challenges necessitate robust and adaptive XRF spectra processing techniques to extract the XRF signal in real time.

**Fig. 1:**
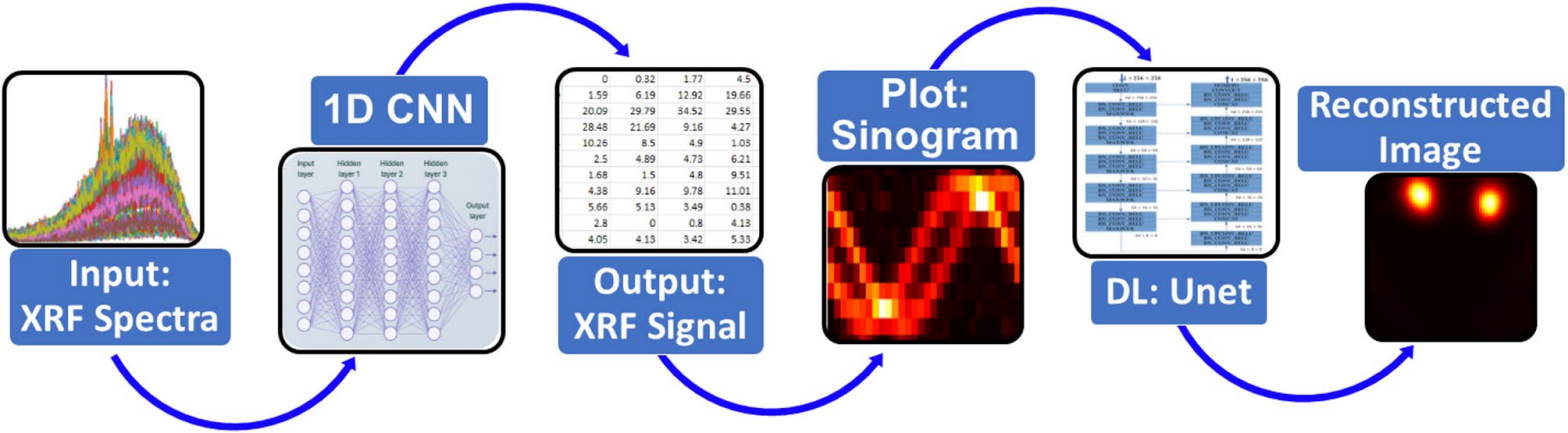
Proposed end-to-end deep learning (DL) framework for XRF signal extraction and image reconstruction. The architecture incorporates a 1D convolutional neural network (CNN) followed by a 2D U-Net model.

Inspired by the great potential of DL algorithm, we propose a novel end-to-end DL framework that integrates a 1D CNN for XRF signal extraction and a 2D U-Net model for image reconstruction (Fig. 1). In this approach, first raw XRF/scatter spectra are processed through a 1D CNN to isolate the XRF signal. Here, XRF/scatter spectra refer to the combined signal comprising both XRF photons and Compton scattered photons, which are not inherently separated in the measured spectrum. The extracted XRF signal is subsequently transformed into sinograms, providing essential data for the subsequent stage. A 2D U-Net model is then applied to produce the reconstructed image from this sinogram. Our method aims to learn complex representations of the raw XRF/scatter spectra directly, bypassing the need for traditional lengthy preprocessing steps. Also, this approach has the potential to address the challenges of removing Compton background noise and attenuation correction in XRF signal. By learning complex data representations, CNN models can effectively disentangle overlapping Compton scatter noise and correct for attenuation. This can lead to more accurate elemental quantification and improved image quality. By jointly optimizing the signal extraction and image reconstruction processes, we anticipate achieving improved performance compared to conventional methods. This integration would significantly advance the practicality and effectiveness of benchtop XFCT for a wide range of applications, making it a more viable tool for routine high-sensitivity quantitative imaging in preclinical application. Additionally, this proposed method can minimize the total imaging dose by reducing the scan time while maintaining adequate image quality for diagnostic/analytical tasks.

## Results

### 1D CNN model

**Fig. 2:**
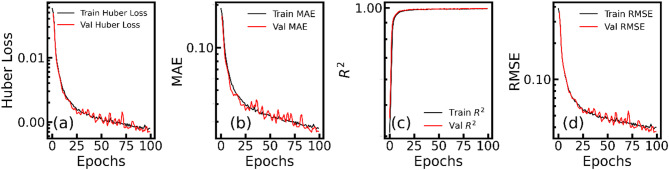
Training and validation performance metrics for the 1D CNN model over epochs (for spectrum level). Subplots (**a**–**d**) illustrate the evolution of Huber loss, mean absolute error (MAE), coefficient of determination (R^2^), and root mean squared error (RMSE), respectively, for both training and validation datasets.

The training was conducted to proceed for a maximum of 100 epochs, incorporating an early termination mechanism depending on validation loss performance. The procedure was configured to terminate training if no improvement in validation loss was observed for 20 epochs, at which point the optimal best weights, corresponding to the lowest validation loss, would be reinstated, helping to prevent overfitting. This model training was completed at epoch 100 (Fig. 2), with the optimal validation Huber loss recorded at 6.690 × 10^−4^ during epoch 92, accompanied by a marginally lower training loss of 8.230 × 10^−4^. The reinstated best weights from this epoch were utilized for all subsequent predictions. A comprehensive evaluation of the model’s performance yielded exceptionally low mean absolute error (MAE) values of 2.809 × 10^−2^ for training and 2.478 × 10^−2^ for validation. The coefficient of determination (R^2^) reflected high reliability in predictions with values of 0.9927 for training and 0.9956 for validation, indicative of the model’s excellent fit to the data. Additionally, the model demonstrated commendable precision, as reflected by root mean squared error (RMSE) values of 4.056 × 10^−2^ for training and 3.564 × 10^−2^ for validation, reinforcing the model’s predictive accuracy. Table 1 highlights all training and testing results.

**Table 1:** Performance metrics of the 1D CNN model across different datasets. The table presents the results for training, augmented test data, and three independent test cases: 3 H Phantom, mouse kidney, and mouse tumor. Metrics include the coefficient of determination (R²), root mean square error (RMSE), Huber loss, and mean absolute error (MAE).

Metrics	Training	Test-Aug data	Test1-3 H phantom	Test2-mouse kidney	Test3-mouse tumor
R^2^	0.9964	0.9959	0.9885	0.9943	0.9001
RMSE	0.8691	0.8916	0.8441	0.6882	0.5100
Huber	0.2377	0.2418	0.2536	0.1815	0.1120
MAE	0.5950	0.6011	0.6248	0.5086	0.3895

During the training phase, the model achieved an impressive R² value of 0.9964. This high value indicates a strong fit to the training data. When evaluated on augmented test data, the R² slightly decreased to 0.9959, still demonstrating the model’s robustness and ability to generalize to new, similar conditions effectively. For the three distinct test scenarios—Test1-3 H Phantom, Test2-Mouse Kidney, and Test3-Mouse Tumor—the model’s R² values were 0.9885, 0.9943, and 0.9001, respectively. These results indicate substantial predictive capability, even though the complexity and diversity of the test datasets resulted in slightly lower R² values compared to the training and augmented test data. The drop in R² for Test3-Mouse Tumor to 0.9001 reflects the increased difficulty of this particular dataset of very low noisy XRF signal. The RMSE values showed a slight increase from 0.8691 during training to 0.8916 on augmented test data. This minor increase suggests that the model still maintains high accuracy on similar new data. For the specific test scenarios, the RMSE values were 0.8441 for Test1-3 H Phantom, indicating better performance compared to training, 0.6882 for Test2-Mouse Kidney, showing further improvement, and 0.5100 for Test3-Mouse Tumor, demonstrating the best performance among the test scenarios. The decrease in RMSE for these test scenarios indicates that the model performs exceptionally well on these specific datasets. Furthermore, Huber loss increased marginally from 0.2377 during training to 0.2418 on augmented test data. The augmented data included baseline shifts, slope shifts, multi-shift scaling, random peak shifts, and Gaussian noise addition to introduce realistic spectral variability. This small increase indicates that the model is still robust against outliers in new data. For the test scenarios, Huber loss values were 0.2536 for Test1-3 H Phantom, slightly higher than augmented data, 0.1815 for Test2-Mouse Kidney, and 0.1120 for Test3-Mouse Tumor. The lower Huber loss values for Test2 and Test3 suggest that the model is particularly effective at handling outliers in these unseen datasets. The MAE increased slightly from 0.5950 in training to 0.6011 on augmented test data. The MAE values for the test scenarios were 0.6248 for Test1-3 H Phantom, slightly higher than the augmented test data, 0.5086 for Test2-Mouse Kidney, and 0.3895 for Test3-Mouse Tumor. The lower MAE values for Test2 and Test3 indicate improved accuracy in these scenarios compared to training and augmented test data. Despite the inherent variability in unknown test datasets, the results underline a notably strong predictive accuracy that persists beyond the training environment. These findings compellingly suggest that the DL model not only excels in a controlled training environment but also retains a significant degree of its predictive accuracy when confronted with new and variable data. This stable performance on previously unseen data highlights the model’s robustness and underlines its potential applicability in diverse XRF signal extraction scenarios.

**Fig. 3:**
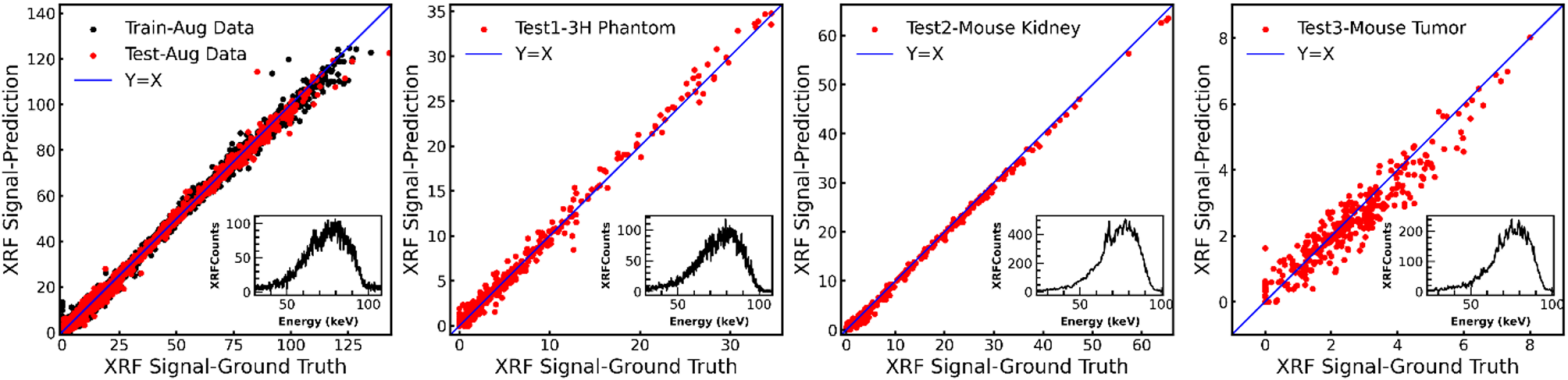
Comparison of XRF signal predictions versus ground truth across different datasets using the 1D CNN model. Each subplot presents a scatter plot of predicted versus ground truth XRF signal values, with the blue line representing the ideal Y = X line. Insets show representative example spectra for each case.

Figure 3 presents the predictive performance of a DL model used for XRF signal extraction. It is apparent that the model performs best on data similar to what it was trained on and experiences a slight decline in performance on the test datasets with Test3-Mouse Tumor data showing the most deviation from the Y = X line particularly in the low signal regime. Despite the observed reduction in precision especially in the case of low signal, the positive correlation of more than 0.90 maintained across all datasets highlights the model’s commendable generalizability and reliability in extracting meaningful signals from previously unseen XRF signal data.

**Fig. 4:**
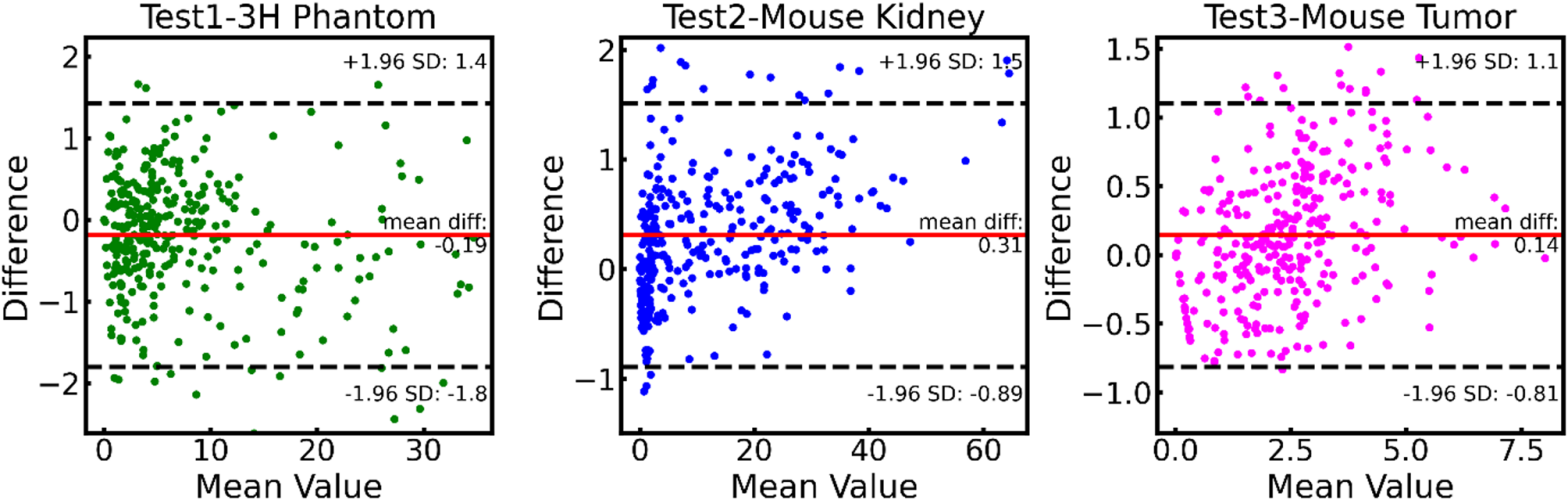
Bland-Altman plots evaluating the agreement between predicted and ground truth XFCT signal values across three independent test datasets. Each plot presents the difference between the predicted and actual values against their mean, with the red line indicating the mean difference and the dashed black lines representing the limits of agreement (± 1.96 SD).

The Bland-Altman plots presented in Fig. 4 evaluate the agreement between predicted and ground truth XRF signal values across three independent test datasets: Test1-3 H Phantom, Test2-Mouse Kidney, and Test3-Mouse Tumor. Each plot displays the difference between the predicted and ground truth values (y-axis) against the mean of the predicted and ground truth values (x-axis), providing a measure of prediction error relative to signal magnitude. For the Test1-3 H Phantom data, the mean difference (bias) is −0.19, with limits of agreement (LoA) ranging from − 1.8 to 1.4, indicating tight consistency within a relatively small error margin. The Test2-Mouse Kidney data exhibit a mean difference of 0.31 with slightly higher LoA from − 0.89 to 1.5, suggesting moderate variability in predictive accuracy. The Test3-Mouse Tumor data show a mean difference of 0.14 and LoA from − 0.81 to 1.1, which are similar to Test1 but exhibit a larger scatter of points, reflecting increased variability. The consistent placement of mean difference near zero across all datasets indicates an overall absence of systematic bias in the model predictions, though the spread of data points within each plot are almost within 95% confidence interval.

**Fig. 5:**
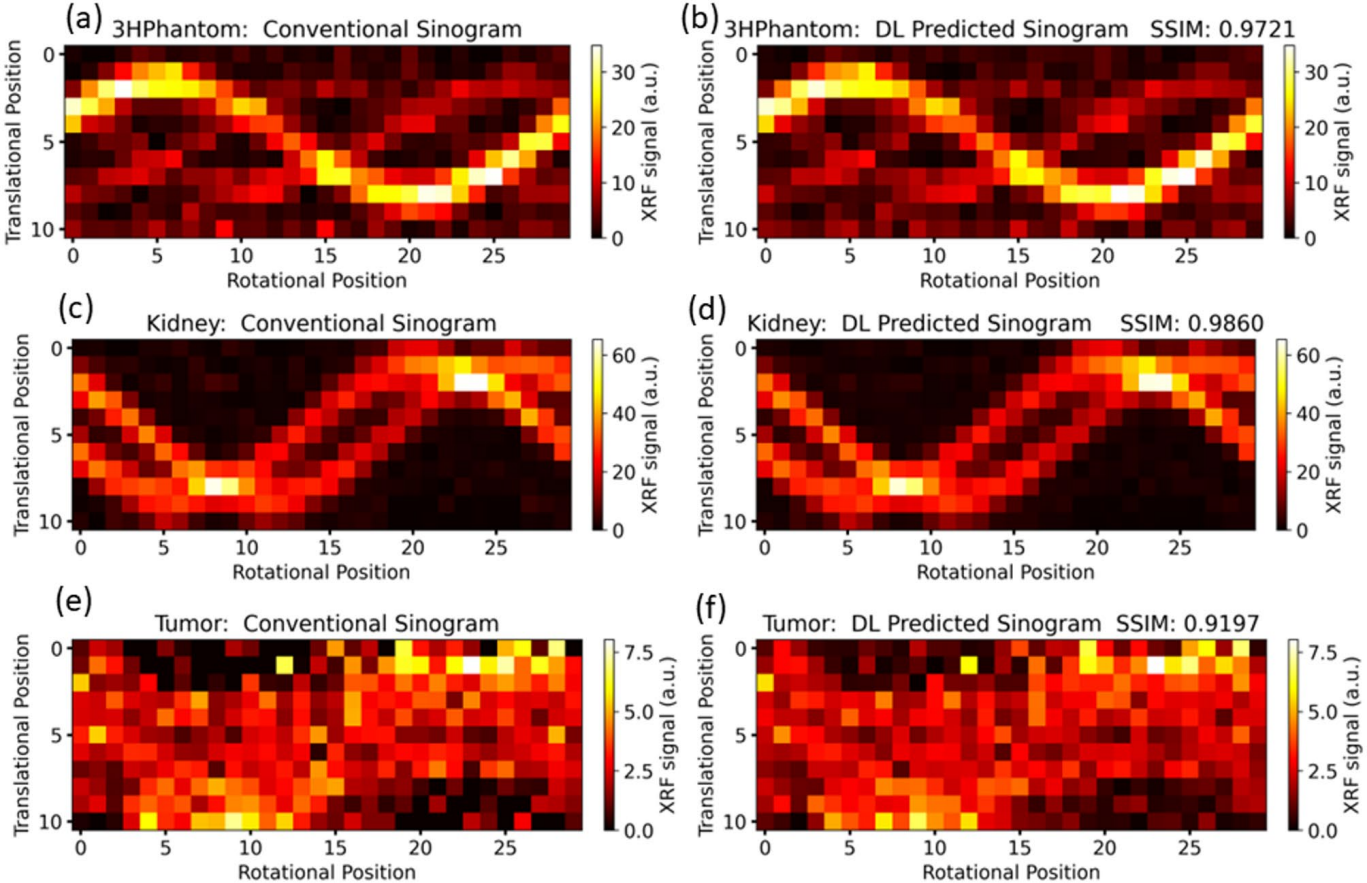
Comparison of conventional sinograms and deep learning (DL) predicted sinograms using a 1D CNN approach for different datasets. Each pair of subplots shows the conventional sinogram (left) and the corresponding DL predicted sinogram (right), along with the Structural Similarity Index (SSIM) for the DL predictions.

Figure 5 compare conventional sinograms with DL predicted sinograms across three different test datasets: 3 H phantom, mouse kidney, and mouse tumor. The SSIM values which quantify the similarity between the conventional and DL predicted sinograms indicate high reliability in the DL predictions. Specifically, the tumor dataset (Fig. 5e, f) shows an SSIM of 0.9197, the mouse kidney dataset (Fig. 5c, d) has an SSIM of 0.9860, and the 3 H phantom dataset (Fig. 5a, b) exhibits an SSIM of 0.9721. These high SSIM values suggest that the DL predicted sinograms closely approximate the ground truth, with minimal loss of structural/anatomical information, particularly in the mouse kidney and 3 H phantom datasets. The slightly lower SSIM for the tumor dataset is expected due to the lower signal intensity in tumor imaging data. Lower XRF signal intensity in the tumor dataset leads to higher noise and weaker contrast, reducing SSIM. Since tumors typically exhibit lower GNP accumulation compared to other organs (e.g., kidneys) imaged by XFCT, structural details are less distinct, making reconstruction more challenging. Despite this, an SSIM above 90% still indicates a good level of similarity, demonstrating that the DL model can effectively extract the signal even in challenging low-signal conditions.

### 2D Unet model

**Fig. 6:**
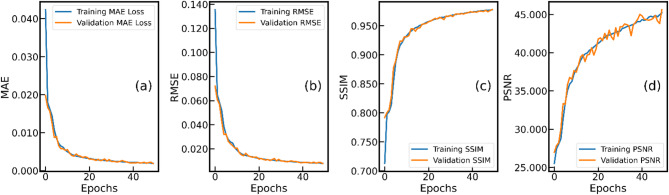
Training and validation performance metrics for the U-Net model over epochs. Subplots (**a**–**d**) illustrate the evolution of Mean Absolute Error (MAE), Root Mean Squared Error (RMSE), Structural Similarity Index (SSIM), and Peak Signal-to-Noise Ratio (PSNR), respectively, for both training and validation datasets.

Figure 6 shows the training and validation performance metrics over epochs for a U-Net model. The MAE for both training and validation datasets rapidly decreases in the initial epochs, indicating a swift improvement in the model’s prediction accuracy. The curves stabilize after epoch 35–40, suggesting the model has effectively learned the underlying patterns and further training shows minimal gains. Both training and validation MAE curves are closely aligned, which implies that the model is not under/overfitting. The RMSE, another measure of prediction error, shows a similar trend to MAE, with a steep decline in the initial epochs and stabilization thereafter. The SSIM values increase rapidly during the initial training phase, indicating improved structural similarity between the reconstructed images and the ground truth. The curves plateau around epoch 35–40, with both training and validation SSIM values nearing 0.9700, which signifies high reliability in the reconstructed images. The PSNR values, which measure the quality of the reconstructed images, show a sharp increase initially, indicating a reduction in noise and an improvement in image quality. There is a fluctuation in the validation curve which are expected due to the inherent variability and limited augmented datasets. Overall, a close alignment between training and validation metrics suggests robust performance without over/underfitting.

**Fig. 7:**
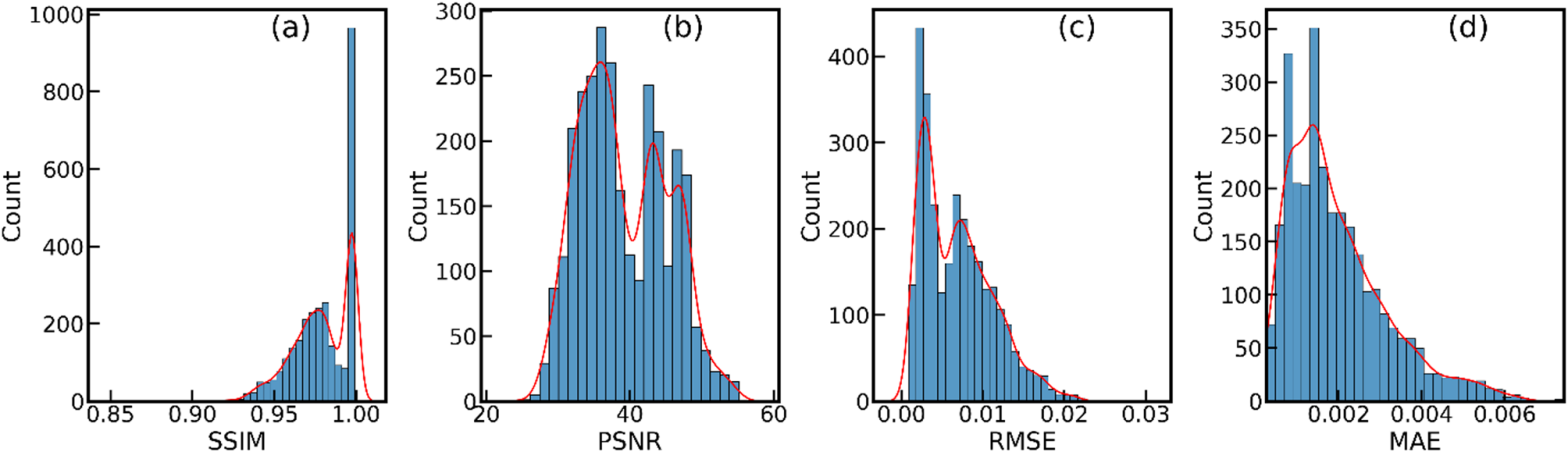
Distribution of image quality metrics on an augmented test dataset (*n* = 2920). (**a**) Structural Similarity Index (SSIM), (**b**) Peak Signal-to-Noise Ratio (PSNR), (**c**) Root Mean Squared Error (RMSE), and (**d**) Mean Absolute Error (MAE). Kernel density estimates (red curve) are overlaid for visualization.

**Fig. 8:**
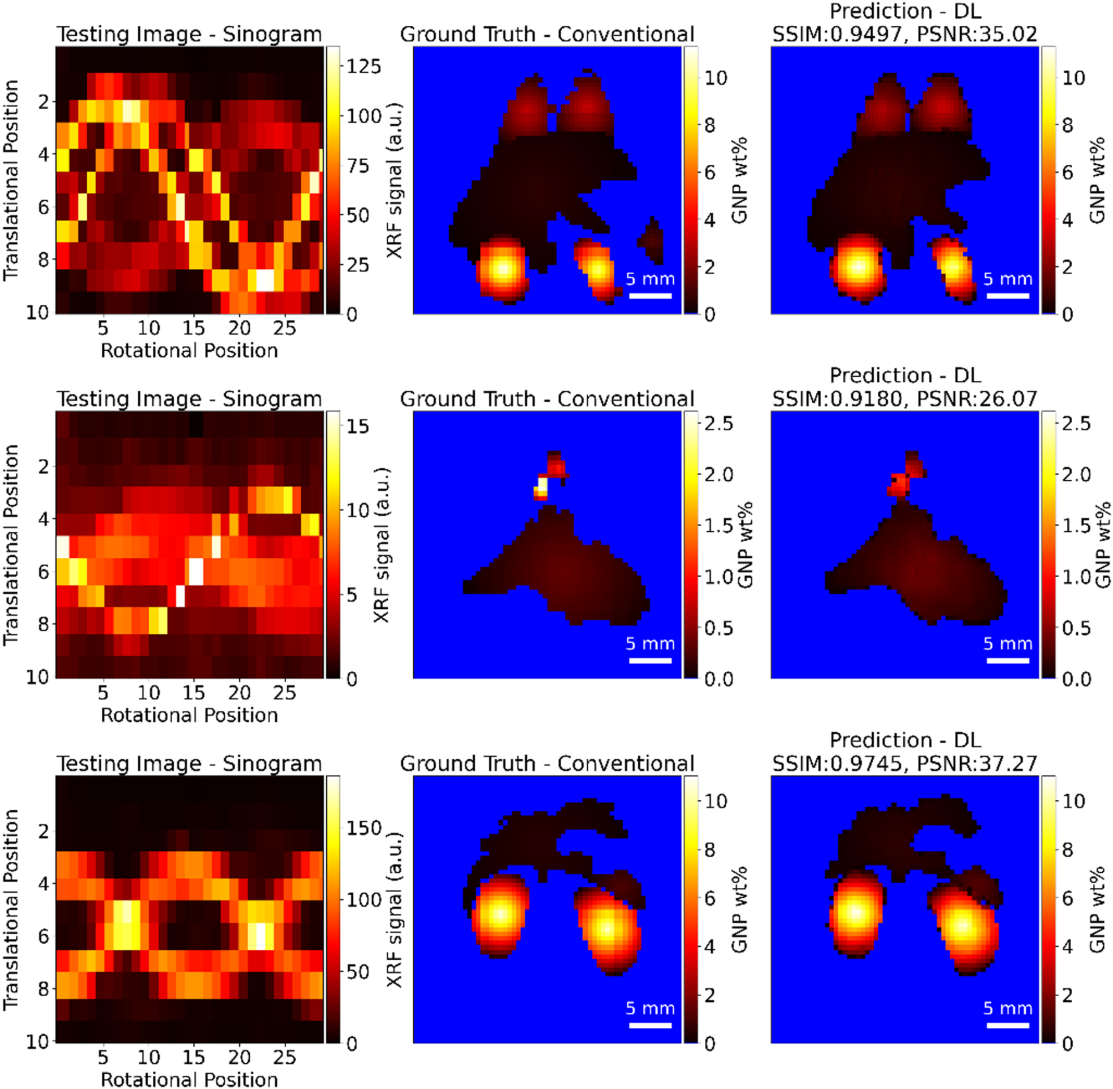
Few examples of reconstructed images from synthetic augmented testing datasets, showing various anatomical regions (heart, lungs, kidney, liver, and spleen) within a mouse phantom. Each row presents a sinogram of the testing image (left), the corresponding ground truth image reconstructed using conventional methods (middle), and the predicted image reconstructed using the deep learning (DL) model (right). Scale bars representing 5 mm are shown in each reconstructed image. To improve the distinction among ground truth, predicted images, and anatomical regions, background concentration values below 0.001 wt% (well below the detection limit) are visually represented in blue.

Figure 7 presents the distribution of four metrics—SSIM, PSNR, RMSE, and MAE—evaluated on an augmented test dataset containing 2920 samples. On average, the test dataset has an average SSIM score of 0.9791, a PSNR of 39.11, an RMSE of 6.900 × 10^−3^, and an MAE of 2.000 × 10^−3^. Figure 7a shows the SSIM values, ranging approximately from 0.8556 to 0.9991. The distribution is right-skewed, with a pronounced peak near 1.0, indicating that most test samples exhibit high structural similarity between the predicted and ground truth data. Figure 7b illustrates the PSNR values, which range from 24.78 to 55.14. The distribution appears bimodal, suggesting that the test samples can be grouped into two distinct sets with differing noise levels or prediction accuracy. This is expected, as the number of projections varied between 30 to 5, with fewer number of projections resulting in larger noise in the images compared to the 30 projections. Notably, even in low-PSNR conditions, the similarity score remains above 90% highlighting the adaptability of the proposed DL approach across varying signal and image quality levels. These cases reflect more realistic imaging scenarios and highlight the robustness of our model in handling high noise. Figure 7c shows the RMSE values, ranging from 8.000 × 10^−4^ to 3.000 × 10^−2^. The distribution is left-skewed, with most values clustered near 7.000 × 10^−3^, signifying that the predictive model generally achieves small errors, though some samples exhibit larger errors. Figure 7d presents the MAE values, with a range from 3.000 × 10^−4^ to 7.500 × 10^−3^. Similar to RMSE, the MAE values are also left skewed, indicating that most test samples have small prediction errors, but a few outliers have larger errors. The KDE plots (Fig. 7), shown in red, provide a smooth estimation of the probability density function for each metric. Overall, the plots indicate that the Unet model performs well on the augmented test dataset, with high SSIM and PSNR values as well as low RMSE and MAE values for most samples. Figure 8 shows few examples of reconstructed images from testing datasets along with their ground truth images.

**Fig. 9:**
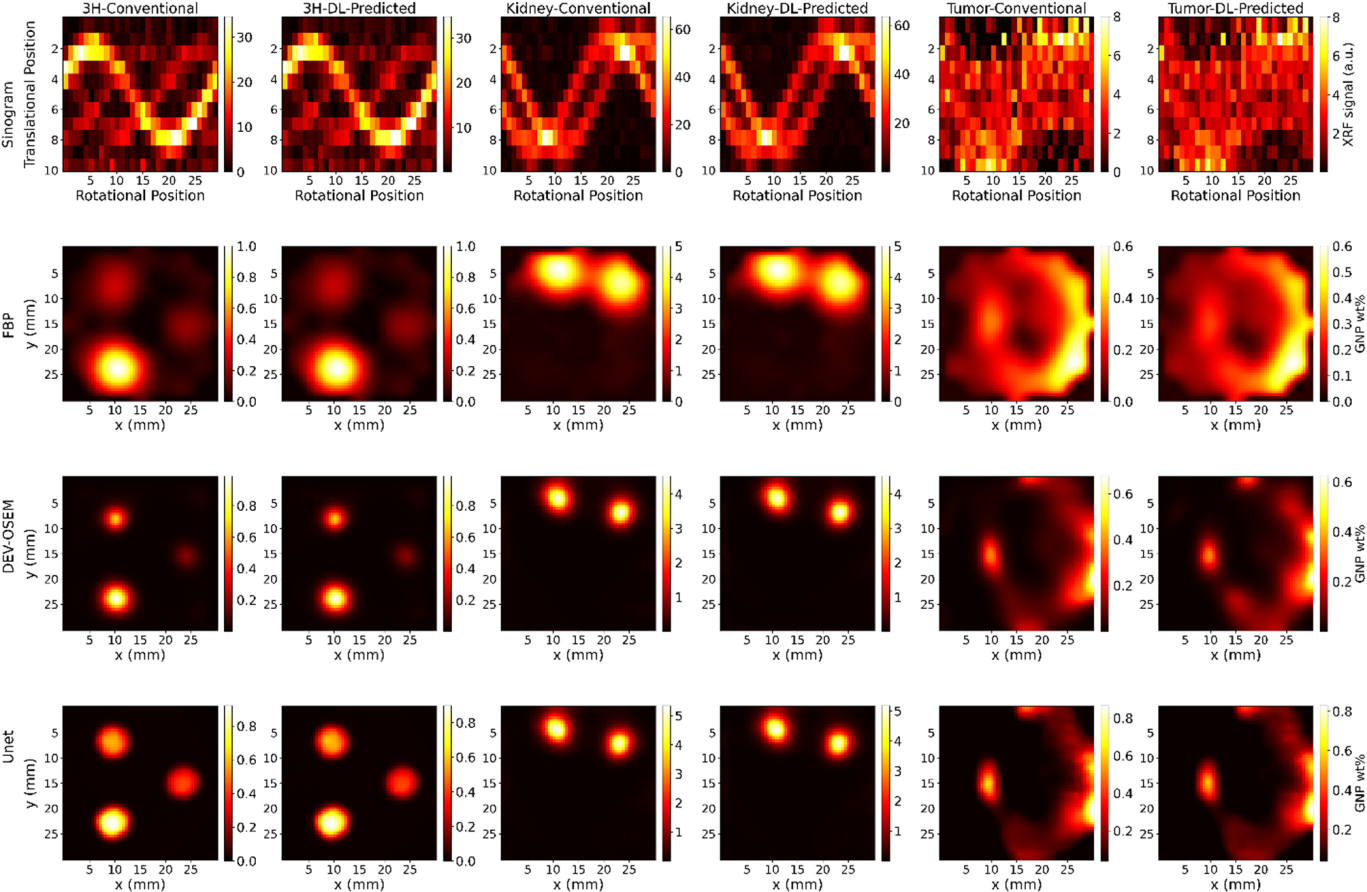
A detailed comparative analysis of reconstructed images using three distinct reconstruction methods: Filtered Back Projection (FBP), DEV-OSEM, and a DL-based U-Net method. Reconstructions were performed on sinograms obtained using both conventional and DL-based XRF signal extraction methods across three test cases: 3 H, mouse kidney, and mouse tumor. The first row shows the sinograms. The second, third, and fourth rows show the reconstructed images using FBP, DEV-OSEM, and the U-Net method, respectively.

Figure 9 presents a detailed comparative analysis of reconstructed images using three distinct reconstruction methods: filtered back projection (FBP), detector’s eye view (DEV)-OSEM^24^, and a DL-based U-Net method. Reconstructions were performed on sinograms obtained using both conventional and DL-based XRF signal extraction methods across three test cases: 3 H, mouse kidney, and mouse tumor. The U-Net method consistently outperformed FBP and DEV-OSEM, offering superior noise reduction and overall image quality, while preserving anatomical details with minimal artifacts. In the reconstructed images of the mouse kidneys, both kidneys were distinctly separated when using DEV-OSEM and the DL model, a notable improvement over FBP. Mouse tumor images, however, exhibited higher noise levels compared to the 3 H and mouse kidney images. This increased noise was attributed to the lower concentration of GNPs in the tumor (0.4 wt%) relative to the other cases (kidney: 4.9 wt% and 3 H phantom: 1 wt%), highlighting the challenge of maintaining image quality at reduced GNP concentrations.

**Fig. 10:**
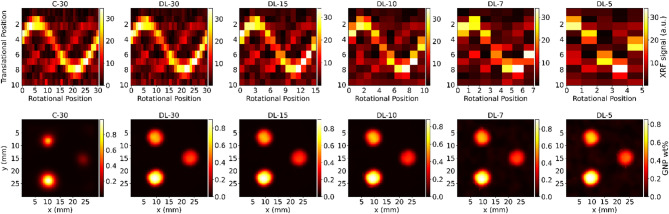
A comparative analysis of conventional and DL-based reconstruction methods for a 3 H phantom containing varying GNP concentrations (1, 0.5, and 0.3 wt%). The top row displays sinograms, while the bottom row showcases corresponding reconstructed images. The first column represents conventional reconstruction using 30 projections, followed by DL-based reconstructions utilizing 30, 15, 10, 7, and 5 angular projections, respectively.

Figure 10 demonstrates the effectiveness of DL models in reconstructing images from TestData1, featuring a 3 H phantom with GNP concentrations of 1, 0.5, and 0.3 wt%. The DL-30 model produced clearer images, outperforming the conventional method. Even with a reduced number of projections, DL models maintained comparable image quality and accuracy. However, at very low numbers of projections (DL-7 and DL-5), noise increased, slightly reducing image quality. In the reconstructed images of the 3 H phantom, the DL method effectively preserved the signal within the 6 mm diameter hole, even at lower GNP concentrations. In contrast, conventional reconstruction methods resulted in a noticeable reduction in both the signal and the apparent diameter of the hole as GNP concentration decreased. This distinction is clearly reflected in the quantification of GNP weight percentages (Table 2), where the DL method shows more accurate and consistent measurements across varying GNP concentrations. Our DL pipeline includes the two key components that preserve signal values and spatial details in GNP-loaded holes. First, the 1D CNN extracts net XRF signals from raw spectra by learning background removal and peak separation without manual preprocessing, thereby improving the signal-to-noise ratio. Second, the 2D U-Net utilizes multi-scale feature extraction and skip connections, which help maintain the spatial integrity and intensity levels of small features, even under conditions with fewer projections or lower GNP concentrations. This robust performance of DL models, even with fewer projections, highlights their potential for reducing radiation exposure while ensuring precise and reliable GNP quantification in preclinical imaging applications.

**Fig. 11:**
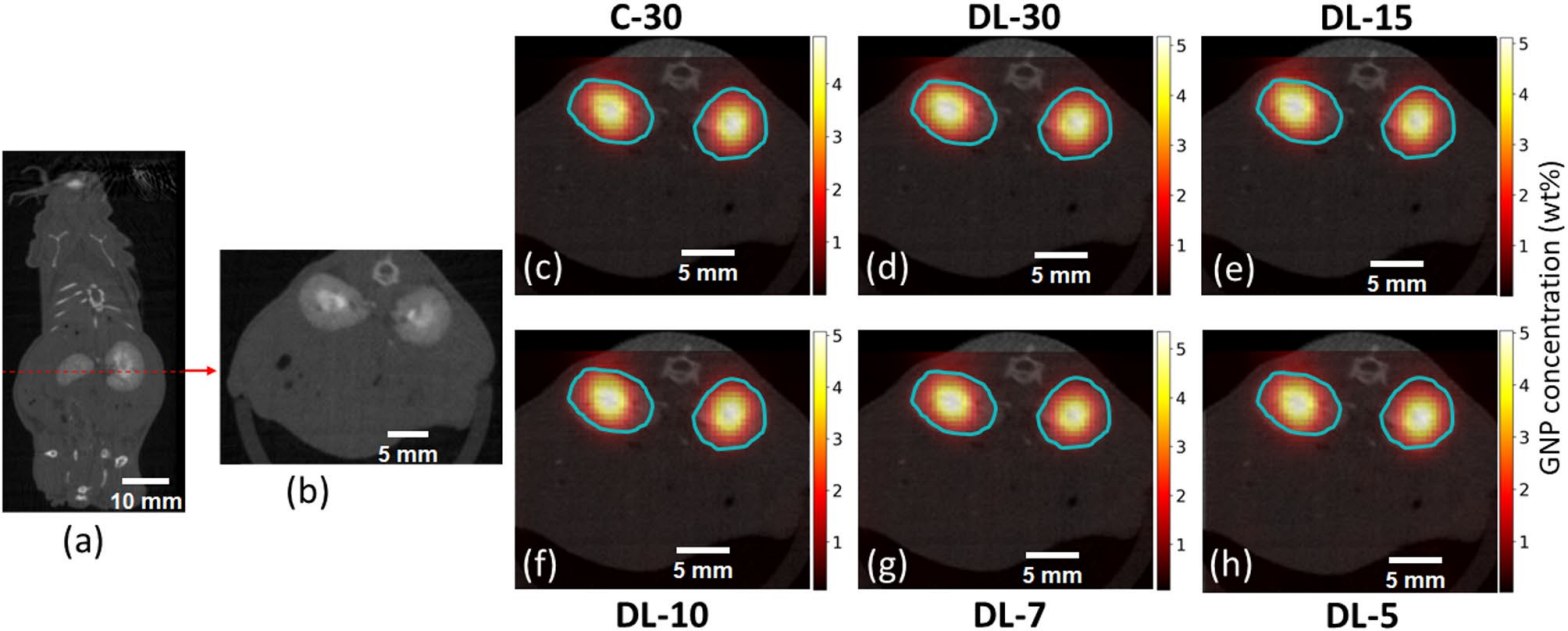
(**a**) Coronal CT slice of a postmortem mouse at 15 min after injection of GNPs. (**b**) Axial CT slice along the dotted line showing left and right kidneys. The displayed images (**c**–**h**) demonstrate the comparative effectiveness of conventional and DL-based methods in reconstructing XFCT images and overlay with CT slices. Image (**c**) represents a conventional reconstruction using 30 angular projections. Images (d-h) represent the innovative DL-based method, applying varied numbers of angular projections − 30, 15, 10, 7, and 5 respectively. For each angular projection there are 11 translation positions. Scale bars representing (**a**) 10 mm and (**b**–**h**) 5 mm are shown in each image.

**Fig. 12:**
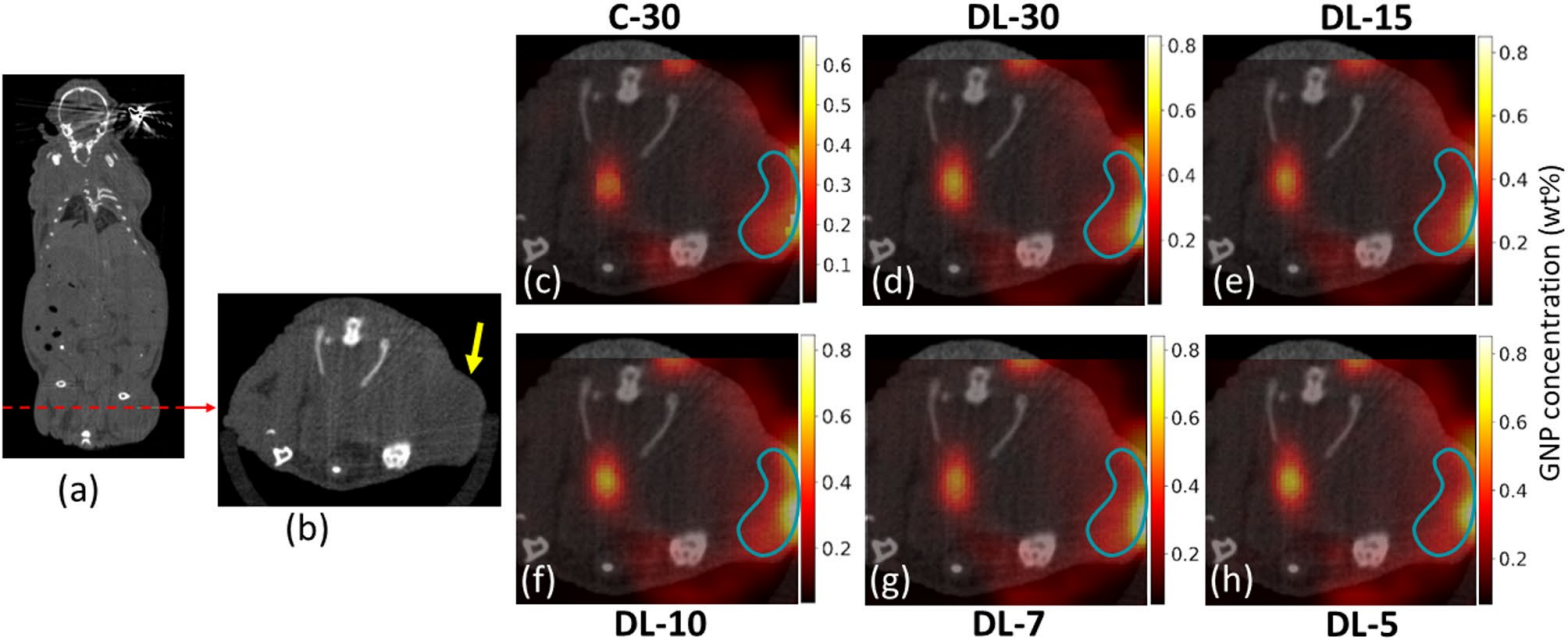
(**a**) Coronal CT slice of a postmortem mouse at 15 min after injection of GNPs. (**b**) Axial CT slice along the dotted line showing tumor region (yellow arrow). The displayed images (**c**–**h**) demonstrate the comparative effectiveness of conventional and DL-based methods in reconstructing XFCT images and overlay with CT slices. Image (**c**) represents a conventional reconstruction using 30 angular projections. Images (**d**–**h**) represent the innovative DL-based method, applying varied numbers of angular projections − 30, 15, 10, 7, and 5 respectively. Scale bars representing (**a**) 10 mm and (**b**–**h**) 5 mm are shown in each image.

Figures 11 and 12 show the results from TestData2 and TestData3, which involved imaging a mouse kidney and tumor regions at 15 min after the injection of GNPs. We compared conventional reconstruction methods using 30 projections (C-30) with DL models using 30, 15, 10, 7, and 5 angular projections. The images demonstrate that the DL-30 and DL-15 models provided superior reconstructions, maintaining high clarity and detail in both the kidney and tumor regions. Notably, the DL-10 model for the kidney produced the results comparable to the conventional C-30 method, indicating the robustness of the DL approach even with fewer projections. In the case of the kidney, as the number of projections decreased further, the DL models continued to maintain high image quality; however, DL-7 and DL-5 showed a slight reduction in clarity and an increase in noise, particularly outside the kidney region. For the tumor case, the images started to become noisier from DL-10, with noise further increasing for DL-7 and DL-5 models. In this study, we compared GNP concentrations derived from a single XFCT slice with the overall GNP content measured by ICP-MS for the excised organ. We acknowledge that biological heterogeneity can lead to spatial variations in GNP accumulation, which may introduce discrepancies between the slice-based XFCT estimation and the whole-organ ICP-MS value. To reduce such discrepancies, we selected a representative slice that captured the primary region of GNP uptake within the organ and, during the XFCT-based quantification, computed mean GNP concentrations within a defined region of interest in the slice. Quantification of GNP concentrations (Table 2) corroborate these findings, with DL-30 and DL-15 models yielding more accurate and consistent measurements compared to the conventional method. For instance, the DL-30 model quantified the GNP wt% in the kidney as 5.103 ± 0.7736, closely matching the inductively coupled plasma mass spectrometry (ICP-MS) result of 4.920 wt%. Similarly, for the tumor, the GNP concentration measured by the DL-30 model was 0.3596 ± 0.0155 wt%, compared to the conventional method’s 0.3121 ± 0.01460 wt% and closely aligning with the ICP-MS result of 0.3900 wt%. The DL-15 model also provided a similar level of accuracy with a quantification of 0.3698 ± 0.01550 wt%. The DL-10 model showed comparable quantification accuracy with a GNP concentration of 0.3774 ± 0.02380 wt%, while DL-7 and DL-5 resulted in slightly lower precision, with measurements of 0.3575 ± 0.02870 wt% and 0.3466 ± 0.02910 wt%, respectively. Notably, our previous study reported GNP concentrations of 4.21 wt% in the kidney and 0.46 wt% in the tumor using conventional peak fitting for XRF signal extraction and FBP for XFCT image reconstruction. While these values are slightly lower as compared to those obtained in the current study, they further highlight the improved quantitative accuracy and robustness of the DL-based approach demonstrated here. These results indicate that even with fewer projections, DL models maintain high accuracy in GNP quantification, making them effective for reducing radiation exposure (i.e., X-ray imaging dose) while preserving image quality. Overall, the DL method effectively preserves anatomical details and provides accurate GNP quantification, underscoring its potential in preclinical imaging applications.

**Table 2:** Quantification of GNP concentrations using various reconstruction methods across three test datasets: TestData1-3 H (with GNP concentrations of 1.0, 0.5, and 0.3 wt%), TestData2-Kidney, and TestData3-Tumor. The table compares conventional reconstruction using 30 projections (C-30) with DL models using 30, 15, 10, 7, and 5 projections. Results are presented as mean ± standard deviation for each method and dataset.

Reconstruction methods	TestData1- 3 H (wt%)			TestData2-Kidney (wt%)	TestData3-Tumor (wt%)
	1.0	0.5	0.3		
C-30	0.6021 ± 0.1914	0.3758 ± 0.1339	0.1795 ± 0.04470	4.566 ± 0.7439	0.3121 ± 0.01460
DL-30	0.7400 ± 0.1616	0.4295 ± 0.07120	0.3103 ± 0.05400	5.103 ± 0.7736	0.3596 ± 0.01550
DL-15	0.6666 ± 0.1461	0.4246 ± 0.06990	0.2997 ± 0.04840	4.965 ± 0.7658	0.3698 ± 0.01550
DL-10	0.6621 ± 0.1450	0.4333 ± 0.07460	0.2823 ± 0.05080	4.918 ± 0.7663	0.3774 ± 0.02380
DL-7	0.6600 ± 0.1418	0.4282 ± 0.06990	0.3095 ± 0.04580	4.9820 ± 0.7769	0.3575 ± 0.02870
DL-5	0.6768 ± 0.1365	0.4471 ± 0.07790	0.3224 ± 0.05250	4.7657 ± 0.7551	0.3466 ± 0.02910

## Discussion

In this study, we developed a complete DL model aimed at enhancing the extraction of XRF signal and reconstruction of benchtop XFCT images. The proposed method leverages a 1D CNN to learn complex data representations directly from raw XRF/scatter photon spectra, bypassing the need for traditional postprocessing steps such as Compton background noise removal and attenuation correction. Our results suggest that the DL model significantly improves elemental quantification accuracy and image quality, providing a more practical tool for real-time high-sensitivity benchtop XFCT imaging in preclinical research. One of the key advantages of our approach is its ability to handle the complexities in raw XRF/scatter photon spectra that include overlapping Compton scatter noise and varying levels of GNP concentrations. By jointly optimizing XRF signal extraction and XFCT image reconstruction, our DL model achieved superior performance compared to conventional methods utilizing traditional approaches (e.g., fitting of raw XRF/scatter photon spectra) for XRF signal extraction and FBP or DEV-OSEM for XFCT reconstruction, as evidenced by high coefficients of determination and low MAE across various test scenarios. Notably, the model maintained high predictive accuracy even when evaluated on completely new unseen experimental datasets with different noise levels and GNP concentrations, underscoring its robustness and generalizability.

**Table 3:** The table highlights the significant advantages of using DL methods over conventional approaches for XFCT in terms of scan efficiency and X-ray dose reduction. It compares the total scan time and the total imaging dose for different numbers of angular projections (30, 15, 10, 7, and 5) with data acquisition time of 5 s and 10 s per projection.

Angular projection	Total scan time (min)		Total imaging dose (cGy)	
	5 s data acquisition per projection	10 s data acquisition per projection	5 s data acquisition per projection	10 s data acquisition per projection
30	2.50	5.00	53.25	106.50
15	1.25	2.50	26.63	53.25
10	0.83	1.67	17.75	35.50
7	0.58	1.17	12.43	24.85
5	0.42	0.83	8.88	17.75

Another key advantage of the DL method lies in its potential to significantly reduce the total scan time and X-ray dose compared to conventional techniques. Tables 3 and 4 highlight the significant advantages of using DL methods over conventional approaches for benchtop XFCT in terms of scan efficiency and X-ray dose reduction. The total imaging dose was calculated following the methodology outlined in a recent work conducted with our latest benchtop XFCT system^13,22^, where the dose rate at the isocenter was determined to be 21.3 cGy/min. As detailed in Table 3, reducing the number of angular projections from 30 to just 5 drastically cuts the total scan time and imaging dose. For example, with 5 projections and a 10-second data acquisition time per projection, the total scan time drops from 5.00 min to 0.83 min, and the imaging dose reduces from 106.50 cGy to 17.75 cGy. This reduction is critical for minimizing X-ray imaging dose and expediting the imaging procedure, making benchtop XFCT more practical for routine use in preclinical research (recommended dose ≤ 40 cGy^8,22^. Additionally, Table 4 demonstrates the remarkable speed-up in data post-processing achieved through the DL method. Using Intel Core i5-8259U CPU/2.30 GHz/16 GB RAM computer, for one slice consisting of 330 XRF/scatter photon spectra, the conventional method requires about 355 s (approximately 6 min) for XRF signal extraction and XFCT image reconstruction, whereas the DL method completes the task in just 1.25 s. Specifically, the DL approach reduces XRF signal extraction time from 319.16 s to 1.17 s and XFCT image reconstruction time from 35.90 s to 0.08 s. This dramatic reduction in postprocessing time enables near real-time display of benchtop XFCT images, significantly enhancing the workflow efficiency. These findings emphasize the potential of DL models to enable faster and more accurate benchtop XFCT imaging while reducing the imaging dose.

**Table 4:** Highlighting the substantial acceleration in data post-processing achieved by the proposed DL method. Processing times for XRF signal extraction and XFCT image reconstruction of a single slice (330 XRF/scatter photon spectra) are compared between the conventional and proposed DL approaches.

1 Slice/330 spectra	Time required (s)	
	Conventional method	Proposed DL method
XRF signal extraction	319.16	1.17
Image reconstruction	35.90	0.08
Total	355.06	1.25

While this study presents promising results, several possibilities for improvement are essential to enhance the model’s robustness and generalizability. First, expanding the dataset to cover a broader spectrum of imaging objects (or anatomical sites), GNP concentrations, and imaging conditions would improve the model’s ability to generalize across various scenarios. Additionally, a more exhaustive hyperparameter optimization, especially for the 2D U-Net process, could further refine the model accuracy and performance. Our current approach, which relies on synthetic/augmented and limited experimental datasets, carries the risk of overfitting or underfitting the DL model in future. To address this, future iterations of our DL model should be trained on more realistic experimental data or data obtained from Monte Carlo (MC) simulations of our benchtop XFCT system and our digital mouse model^41^, as well as other digital mouse models. This approach, which includes various scenarios of mouse imaging where the XRF/scatter photon profiles may vary despite anatomical similarities, will help generalize the predictions. Given the short training time required (approximately 10 min for 1D CNN and 2 h for U-Net), new complex cases can be quickly added to the model for retraining, which aims to adapt the model to new data and provide more accurate and up-to-date results using existing parameters. If the image acquisition geometry changes (e.g., variations in angular sampling, detector type, or beam configuration), adjustments primarily involve reformatting the spectral and positional input data, leaving the core architecture largely intact. In more drastic scenarios—such as shifting from GNPs to a different MNP probe or contrast agent —transfer learning with a new dataset can fine-tune the model. Minimal hyperparameter tuning may sometimes be beneficial, but extensive architectural modifications are typically not necessary especially for 1D CNN because the underlying task (i.e., learning the relationship between raw photon spectra and elemental XRF signal content) remains unchanged. Likewise, in case of the sample shifts (e.g., from mice to rats), retraining on new data may be necessary. Ultimately, our goal is to create an atlas of application-specific benchtop XFCT imaging data for our DL algorithms, enabling real-time spectral data processing and benchtop XFCT image reconstruction. For completely new cases and/or more accurate quantitative imaging, our conventional post-processing approach will still be utilized. This dual approach ensures that we can control the speed and efficiency of DL methods while maintaining the accuracy and reliability of conventional techniques for novel or particularly challenging case. By addressing the aforementioned limitations and pursuing the proposed paths of future research, this powerful imaging modality (i.e., benchtop XFCT) can be further refined and applied to a wider range of applications. The demonstrated ability to extract meaningful information from complex XRF/scatter photon spectra and reconstruct high-quality XFCT images holds immense potential for advancing both preclinical and clinical research.

## Conclusions

In this study, we developed and validated an advanced end-to-end DL framework that significantly improves the efficiency and accuracy of benchtop XFCT imaging for preclinical applications. By integrating a 1D CNN for real-time XRF signal extraction with a 2D U-Net model for image reconstruction, our approach addresses the limitations of conventional methods that involve time-consuming complex data post-processing procedures. The DL models demonstrated remarkable performance, achieving a high coefficient of determination and a low mean absolute error in XRF signal extraction across various test scenarios, including challenging low concentration datasets like the mouse tumor with an R² of 0.9001. The 2D U-Net model enabled reconstruction of high-quality images with an average SSIM of 0.9791 and a PSNR of 39.11, closely matching the ground truth. Notably, the total data post-processing time per slice was reduced from approximately 6 min using conventional methods to just 1.25 s with our DL framework—a reduction of over 99%. Additionally, the DL models maintained high image quality even when the number of angular projections was reduced from 30 to as few as 5, resulting in a significant decrease in imaging dose. Furthermore, our method provided accurate quantification of GNP concentrations in critical biological tissues using fewer projection; for instance, in the mouse kidney dataset, the DL model measured 5.10 ± 0.77 wt% with just 10 projections, closely aligning with the ICP-MS result of 4.92 wt%, whereas the conventional method required 30 projections and reported 4.57 ± 0.74 wt%. In the mouse tumor dataset, the DL model estimated a GNP concentration of 0.36 ± 0.02 wt% using only 10 projections, compared to 0.31 ± 0.01 wt% by the conventional method, and closely matching the ICP-MS result of 0.39 wt%. These findings underline the efficacy of our DL approach in maintaining high image quality and quantification accuracy even with as few as 5–10 projections. By overcoming key limitations of conventional methods, our DL-enhanced framework brings us closer to achieving near real-time, high-sensitivity benchtop XFCT imaging. This advancement not only boosts the capabilities of benchtop XFCT but also lays a solid foundation for future research exploring the potential of DL in benchtop XFCT imaging.

## Methods

To ensure robust training and accurate reconstruction, a dual-level training strategy was employed, wherein the 1D CNN model was trained at the photon spectrum level, while the 2D U-Net model was trained at the sinogram level. This hierarchical approach effectively extracts spectral and spatial features, enhancing the reliability of XFCT image reconstruction.

### Datasets for 1D CNN

**Fig. 13:**
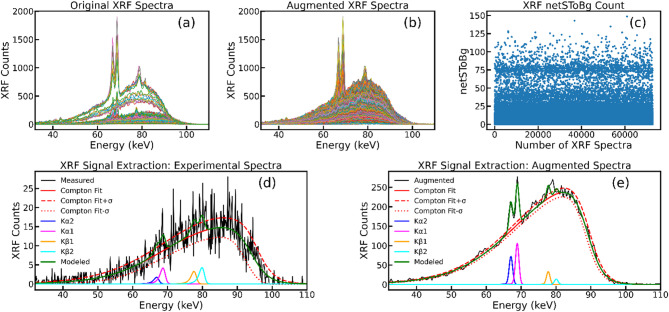
An overview of the dataset and preprocessing steps. (**a**) Representative original XRF/scatter spectra. (**b**) Augmented XRF spectra generated to expand dataset diversity. (**c**) Scatter plot of net signal-to-background ratio (netSToBg) for augmented spectra, demonstrating signal quality distribution. (**d**) Deconvolution-based XRF signal extraction from a 0.1 wt% GNP sample, illustrating measured (experimental) data, Compton fit, and extracted gold K-shell XRF peaks. (**e**) Deconvolution-based XRF signal extraction from an augmented sample spectrum.

A detailed description of the experimental benchtop XFCT system employed for data acquisition can be found in our previous studies^11,13,42^. Briefly, our latest benchtop XFCT system includes a COMET MXR-160/22 tungsten target X-ray source, operating at 125 kVp and 24 mA with a 5.5-mm focal spot size and producing a 1.8-mm Sn-filtered cone beam (~ 3 cm diameter at the isocenter). The source-to-isocenter distance is 15 cm, while the isocenter-to-detector distance is 10 cm. The detector is positioned at 90° to the beam direction, placed behind a parallel-hole collimator to optimize signal detection. Adhering to this experimental setup, a total of 144 XRF/scatter spectra were initially collected using a pixelated CdTe detector system, known as HEXITEC (Quantum Detectors, Inc., Oxfordshire, UK), for varying concentrations of GNPs ranging from 0.01 to 1.0 wt% suspended in phosphate-buffered saline (PBS) within 12-mm-diameter plastic tube phantoms. To introduce varying levels of noise, spectra were acquired at acquisition times of 5, 10, 20, and 60 s. Similarly, an additional 144 XRF/scatter spectra were collected using a compact, thermoelectrically cooled single-crystal CdTe detector (AXR-CdTe; Amptek Inc., Bedford, MA) for a range of GNP concentrations. To establish a baseline, 56 spectra without GNPs were also acquired. To extend the dataset’s applicability to more complex scenarios, 10 randomly selected spectra were collected from a 3 cm-diameter, 3 cm-height, polymethyl methacrylate (PMMA) cylindrical phantom containing three GNP-loaded cylindrical holes (6 mm diameter, 15 mm depth), called 3 H-phantom, using the HEXITEC detector. Each hole contained a different GNP concentration (0.1, 0.3, and 0.5 wt%). For the HEXITEC detector, with a 2 cm × 2 cm field of view (FOV), a single horizontal translation was performed to fully cover the 3 cm imaging phantom, while no translation was required for phantoms smaller than FOV. Furthermore, 10 randomly sampled spectra from a tumor-bearing mouse, previously obtained in our postmortem animal imaging study^11^, were also included. In this case, for the single-crystal CdTe detector, the detector was translated 10 times, resulting in 11 translational positions with a 3-mm step size, while angular positions were sampled in 12° steps. This expanded dataset, comprising a total of 364 XRF/scatter spectra (Fig. 13a), was subsequently augmented to mitigate the limitations of insufficient training data for the DL model development. To enhance the model’s robustness to real-world data variability, data augmentation was performed by introducing baseline shifts, random noise, and applying random slopes, axis offsets, and multishift procedures to each spectrum using a Python script. Baseline shifts were introduced by adding a random vertical offset to each spectrum which simulate the background variations caused by detector fluctuations. Slope shifts were applied as small intensity gradients along the energy axis to account for spectral drift and variations in detector response. To address minor energy calibration variations, random axis offsets (peak shifts) of ± 1 channel were incorporated by shifting spectral data along the x-axis. Additionally, multi-shift scaling was implemented by multiplying the spectrum by a random scaling factor to mimic detector gain fluctuations. Finally, random noise was added using a Gaussian distribution to replicate detector uncertainties and experimental fluctuations. This comprehensive approach effectively introduced realistic spectral variability while preserving key spectral features and enhancing the model’s ability to generalize across different experimental conditions. This process was repeated 200 times for each of the 364 original spectra, resulting in an augmented dataset of 72,800 spectra (Fig. 13b).

For supervised DL training, paired input-output data are essential. To generate this dataset, acquired XRF/scatter spectra underwent initial correction for CdTe detection efficiency. Subsequently, XRF counts were extracted using a deconvolution-based method outlined in our previous study^23^. XRF counts under gold Kα1 and Kα2 XRF peaks were summed to obtain the net XRF counts as shown in Fig. 13d, e. Consistent with the previous studies, XRF counts of less than 1.96σ (standard deviation of the background at the 95% confidence level) were considered statistically insignificant. Attenuation correction was achieved by calculating the ratio of net XRF signal to Compton scatter background, with Compton scatter serving as an internal attenuation probe^43^. This approach enabled absolute quantification of GNPs within the animal/phantom. The resulting net signal to background (netSToBg) values, which represent the conventional method for XRF signal extraction and subsequent reconstruction, served as the ground truth output for training the DL model (Fig. 13c). Based on these extracted signals, sinograms were constructed by arranging the data at their corresponding angular and translational positions.

The dataset was initially partitioned into training and testing sets following a 70/30 split, resulting in 50,960 and 21,840 spectra, respectively. Subsequently, the training set was further divided into training and validation subsets during the training using an 80/20 ratio (40,768 and 10,192 spectra). For independent evaluation, three additional datasets were created. TestData1 comprised 330 spectra acquired from a 3 H-phantom filled with varying GNP concentrations (1.0, 0.5, and 0.3 wt%). TestData2 and TestData3 consisted of 330 spectra each, focused on kidney and tumor regions from tumor-bearing mice^11^. A global standard scaler derived from the training set using scikit-learn was applied to normalize all datasets. To facilitate result interpretation, data were inverse transformed back to the original range post-training and prediction.

### Dataset for 2D Unet

To train a 2D U-Net model, a synthetic dataset containing 100 sinogram-reconstructed image pairs from a cylindrical 3 H-phantom with varying GNP concentrations and 192 sinogram-reconstructed image data (heart, lungs, kidney, liver, and spleen regions) of a mouse phantom^44^ mimicking a GNP-injected mouse was generated. For mouse dataset, GNP concentrations were distributed in various anatomical regions according to inductively coupled plasma mass spectrometry (ICP-MS) results of our previous study^11^. Using these images, sinograms were generated through the Radon transform. Noise-free reconstructed images served as the ground truth for accurate image evaluation. Our benchtop XFCT system typically employs 30 projections acquired with a 12-degree rotational step size for a complete 360-degree scan. To investigate the potential for reduced scan time and X-ray dose, the number of angular projections was systematically decreased from 30 to as few as five, resulting in a dataset of 5840 sinogram-image pairs. Data augmentation, including multiplication, flipping, rotation, and Gaussian noise addition, was applied to expand the dataset to 29,200 pairs. The data was split 70/20/10% into training (20440 pairs), validation (5840 pairs), and testing (2920 pairs) sets. The same three test data sets used for 1D CNN signal extraction was used for independent testing. A global min-max scaler was applied to normalize the data, and inverse transformation was used to restore the original data range post-processing. During inference, the raw sinogram data for each image were translated into the average GNP concentration within each pixel of the reconstructed XFCT image using the calibration curve^11^ derived under the same imaging geometry. This process was carried out using a conventional method to ensure accuracy and consistency.

### 1D CNN model

The model was built using a Keras sequential framework, comprising a linear stack of layers. Its architecture included two convolutional layers with 75 and 14 filters, and kernel size of 39 and 13, respectively. The activation function employed was the exponential linear unit (ELU). To mitigate overfitting and enhance generalization, the model incorporated a dropout layer with a rate of 0.22 and introduced a small amount of Gaussian noise to the input data. The final layers were fully connected, with the second-to-last layer containing 260 neurons and the output layer providing a single linearly activated unit. The model was compiled with the Huber loss function^45^, which is less sensitive to outliers in the data than the mean squared error loss. It follows MSE for small errors to ensure smooth optimization but shifts to MAE for larger errors, making it more robust to outliers. This makes it well-suited for XRF spectral data and ensures stability in the presence of measurement noise and extreme values. The Adam optimizer with a very low learning rate (5.400 × 10^−5^) was used for gradual weight adjustments. Evaluation metrics included mean absolute error (MAE), root mean squared error (RMSE), and coefficient of determination (R^2^). The input dimension of the model was (500, 1), as only 500 channels (200–700) out of 800 in the XRF spectra, representing a range of 32 keV to 112 keV with a bin width of 0.16 keV, were included. The hyperparameters of the CNN model were implemented and optimized using advanced Bayesian optimization techniques^46^, running for 50 trials over 100 epochs. Parameter values and optimizations are detailed in Table 5. The final training was conducted for 100 epochs on a 16 GB NVIDIA V100 GPU, taking approximately 10 min to complete with a batch size of 128.

**Table 5:** Hyperparameter optimization results for the neural network model. The table lists the hyperparameters, their respective ranges explored during optimization, and the optimized values identified for each parameter.

Hyperparameter	Parameter range	Optimized parameter
Dense layer	{50, 60, 70, …, 750}	260
Dropout rate	U (0.001, 0.5)	0.22
C1_k (Filter in first convolution layer)	{3, 4, 5, …, 100}	75
C1_s (Kernel size in first convolution layer)	{3, 4, 5, …, 50}	39
C2_k (Filter in second convolution layer)	{3, 4, 5, …, 100}	14
C2_s (Kernel size in second convolution layer)	{3, 4, 5, …, 50}	13
Batch size	{64, 128, 256}	128
Learning rate	Loguniform (0.00001, 0.01)	0.000054
Activation function	{relu, elu, tanh}	elu

### 2D Unet model

A modified Keras U-Net architecture, adapted from Żak’s implementation on GitHub^47^, was used for sinogram to image reconstruction tasks. A 256 × 256 sinogram served as input, undergoing multiple convolutional and rectified linear unit (ReLU) layers in the encoder before up sampling back to the original dimensions in the decoder. Skip connections were incorporated to preserve spatial information by concatenating features from the encoder to corresponding decoder layers. Unlike typical U-Net model^36^ that increases feature maps with each max pooling, we maintained a constant 64 feature maps throughout. This was based on the network’s ability to access low-level features in the up-sampling path and the nature of sinogram images, which do not require understanding of very high-level 3D like objects. The resulting model, comprising 1,616,289 parameters, was trained to learn intricate patterns within the data for accurate image reconstruction. The model was compiled using the Adam optimizer with a learning rate of 0.0001 and the MAE loss function. Additionally, the model was evaluated with metrics including RMSE, structural similarity index measure (SSIM), and peak signal-to-noise ratio (PSNR)^48^ The final training was conducted for 50 epochs on a 16 GB NVIDIA V100 GPU, taking approximately 2 h to complete with a batch size of 128.

## Data Availability

The data that support the findings of this study are available from the corresponding author upon reasonable request.
